# Swallow syncope: a case report and review of literature

**DOI:** 10.1186/s12872-019-1174-4

**Published:** 2019-08-07

**Authors:** Kelvin Shenq Woei Siew, Maw Pin Tan, Ida Normiha Hilmi, Alexander Loch

**Affiliations:** 1 Department of Medicine/Cardiology, University Malaya Medical Centre, Kuala Lumpur, Malaysia; 2 Department of Medicine/Geriatric, University Malaya Medical Centre, Kuala Lumpur, Malaysia; 3 Department of Medicine/Gastroenterology, University Malaya Medical Centre, Kuala Lumpur, Malaysia

**Keywords:** Swallow, Syncope, Deglutition, Bradycardia, AV block, Pacemaker

## Abstract

**Background:**

Swallow or deglutition syncope is an unusual type of neurally-mediated syncope associated with life-threatening bradyarrhythmia and hypotension. It is a difficult condition to diagnose with commonly delayed diagnosis and management. There is lack of review articles that elucidate the basic demographics, clinical characteristics and management of this rare condition. This publication systematically reviews the 101 case reports published since 1793 on swallow syncope.

**Case presentation:**

A 59-year-old man presented with the complaint of recurrent dizziness associated with meals. A 24-h ambulatory ECG recording confirmed an episode of p-wave asystole at the time of food intake. Oesophagogastroduodenoscopy with balloon inflation in the mid to lower oesophagus resulted in a 5.6 s sinus pause. The patient’s symptoms resolved completely following insertion of a permanent dual chamber pacemaker.

**Conclusions:**

Swallow syncope is extremely rare, but still needs to be considered during diagnostic workup. It is commonly associated with gastro-intestinal disease. Permanent pacemaker implantation is the first line treatment.

## Background

Swallow syncope is a rare cause of a neurally mediated syncope that is frequently associated with life-threatening bradyarrhythmia [1]. The underlying mechanism is believed to be an exaggerated vagal stimulation during swallowing resulting in suppression of the cardiac conduction system. Swallow syncope has been reported in all age groups and occurs with or without underlying esophageal or cardiac pathology. A diagnosis of swallow syncope is frequently missed by physicians, often resulting in delayed diagnosis and treatment. The first case of swallow syncope was reported by Spens in 1793 [2]. Since then, another 117 cases have been reported in the literature.

We present a case of recurrent swallow syncope with a review and summary of the entire literature available regarding this rare condition.

## Case presentation

**Fig. 1:**
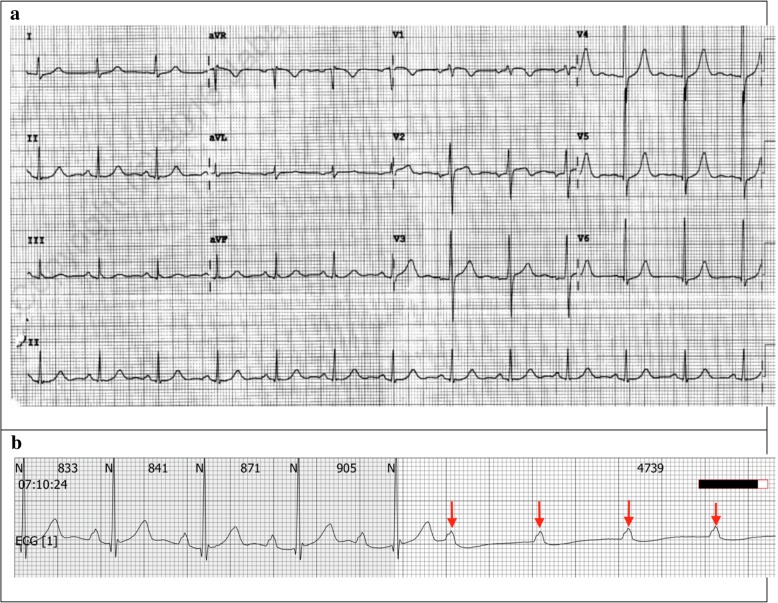
**a** 12-lead electrocardiogram with normal sinus rhythm during non-meal times. **b** 4.5 s episode of non-conducted p-waves during breakfast on a 24-h ECG. Arrow denotes p-waves

A 59-year-old Chinese male presented with a 6-month history of intermittent dizziness. The dizziness occurred exclusively at meal times and was worst when swallowing large quantities of solid food, such as rice or bread. He initially was symptom free when consuming smaller quantities of solids or fluids, but his condition worsened progressively with presyncopal events occurring even while eating smaller quantities of solid food. The patient described a sensation of increasing difficulty in swallowing despite reducing the size of his meals. He denied any associated syncope or seizures. His past medical history and physical examination were unremarkable and blood investigations were within normal limits. Echocardiography revealed a structurally normal heart with normal systolic and diastolic function. 24-h electrocardiogram (ECG) monitoring recorded a sinus pause of 4.5 s at the time the patient had his meal (Fig. 1). A provisional diagnosis of swallow syncope was made and a permanent pacemaker (PPM) implantation was scheduled.

**Fig. 2:**
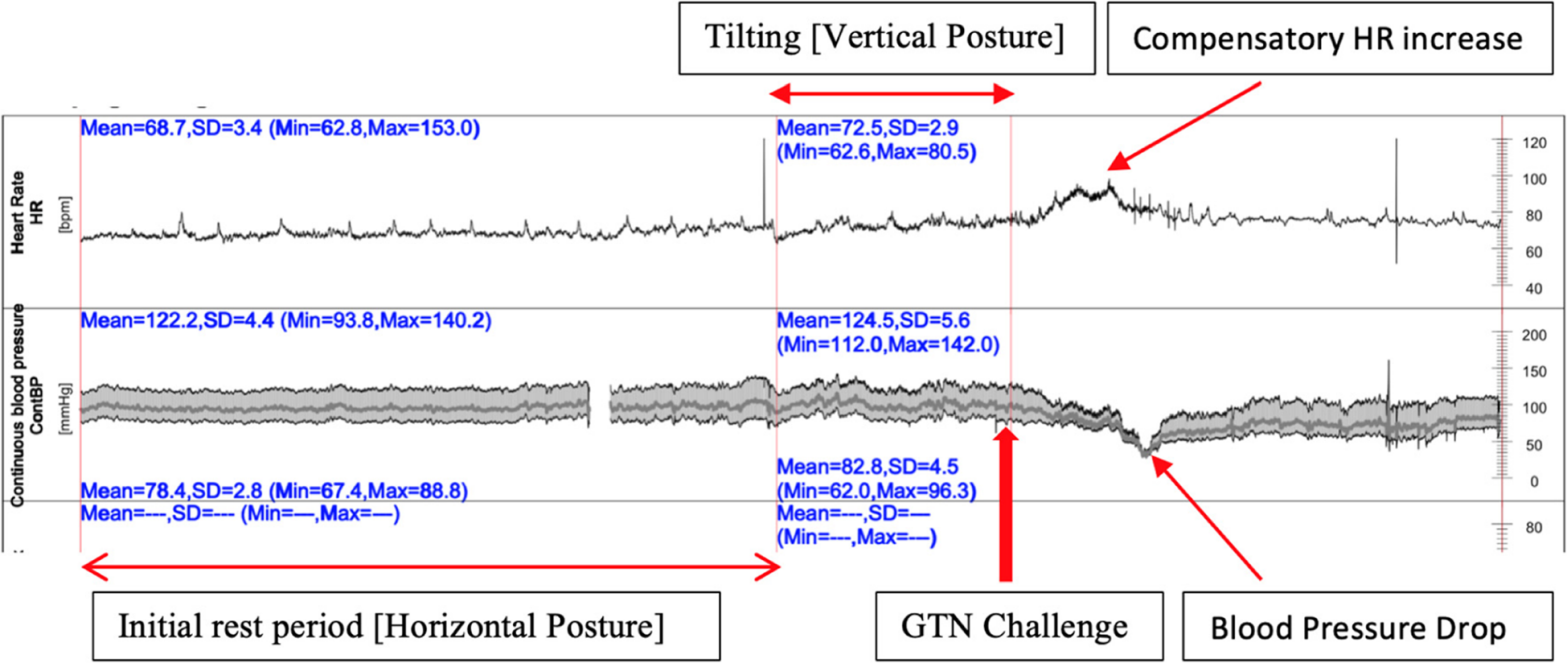
Tilt table test

Tilt table testing prior to pacemaker insertion resulted in a hypotensive response 5 min after provocation with 400 micrograms of sublingual glycerin trinitrate administered sublingually, with reproduction of symptoms of syncope. The minimal blood pressure was 32.9/29.2 mmHg and the heart rate 75.3 bpm. No asystole was observed during tilt table testing (Fig. 2).

**Fig. 3:**
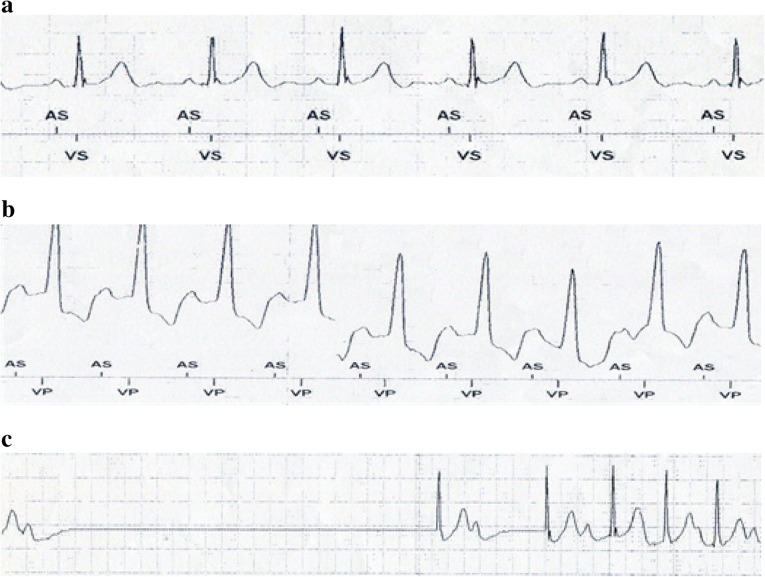
**a** Pacemaker recording of patient in sinus rhythm prior to OGDS procedure, intrinsic heart rate 65 beats/min. **b** Pacemaker recording during advancement of endoscope into distal oesophagus (Pacemaker ON), increasing ventricular pacing. **c** Pacemaker recording when balloon inflation in distal oesophagus (Pacemaker OFF), 5.6 s pause

The patient’s symptoms resolved completely after implantation of a dual chamber PPM. A diagnostic workup to exclude gastrointestinal disease was performed. A barium swallow test was normal and effectively ruled out achalasia. The oesophagus appeared healthy with no structural disease on oesophagogastroduodenoscopy (OGD). The pacemaker was continuously interrogated during the OGD. Increased pacing requirements were noted when the endoscope was advanced into the esophagus (Fig. 3b). Subsequently, a 20 mm diameter TTS (through-the-scope), CRE™ (controlled radial expansion) balloon (Boston scientific) was sequentially inflated in the proximal, mid and distal esophagus while the pacemaker was programmed “OFF” to assess the physiologic response. Inflation in both distal and mid oesophagus resulted in significant sinus pauses of up to 5.6 s (Fig. 3c) confirming the cardio-inhibitory response to oesophageal distension as the underlying pathophysiological mechanism of this patient’s syncopal events.

## Discussion

Swallow syncope is more common in males (59.4%, *n* = 60), and in the older age group (55.4%, *n* = 56, more than 60 years old). The mean age at presentation was 57.5 years with the youngest patient described in the literature being 5 years old [3] and the eldest 89 years old [4]. All of the patient presented with either presyncope or syncope. Only one patient was diagnosed incidentally, when a high degree atrioventricular (AV) block associated with meal times was found during a diagnostic workup for lung carcinoma [5]. Swallow syncope is strongly associated with gastrointestinal diseases (32.7%, *n* = 33). Hiatal hernia (18.8%, *n* = 19), oesophageal stricture (3%, *n* = 3), achalasia (3%, *n* = 3) and oesophageal carcinoma are the most common associated gastrointestinal disorders. Thirty-three patients (32.7%) had underlying cardiac diseases including coronary artery diseases (13.9%, *n* = 14), atrial fibrillation (5%, *n* = 5), sick sinus syndrome (3%, *n* = 3), aortic aneurysm, rheumatic heart disease and digitalis toxicity. Twenty-eight patients (27.7%) had metabolic diseases like hypertension, diabetes mellitus, dyslipidaemia or obesity.

In most patients (54.5%, *n* = 55), any type of food – be it liquids or solids - triggered syncope. Atrioventricular conduction blocks (34.7%, *n* = 35) including first, second and third-degree AV blocks are the most common electrophysiological problems, followed closely by sinus node dysfunctions (33.7%, *n* = 34) including sinus bradycardia, sinus arrest and asystole. Second degree AV block, complete heart block (=3rd degree AV block) and asystole were the most frequently reported bradyarrhythmia in the literature. However, there are several cases where both sinus and atrioventricular dysfunction concurred. Paroxysmal atrial fibrillation and atrial tachycardia were rare causes of syncope. Table 1.

**Table 1:** Literature review of 101 cases of Swallow Syncope from 1949 to 2018

Author/ Reference	Age/ Gender	Presenting Symptom	Underlying Diseases	Trigger Factor	Type of arrhythmia	Management	Effectiveness
Padalia et al. 2018/ [6]	65/ F	Presyncope, Dysphagia, Odynophagia/	Candida Esophagitis, Metabolic Diseases	Solid and Liquid	Sinus bradycardia, Sinus arrest	Micafugin	Yes
Sammy et al. 2018/ [7]	67/M	Syncope	End Stage Renal Failure	Ascension of Hyoid bone irritate carotid sinus	–	–	–
Yamaguchi et al. 2018/ [8]	76/M	Syncope	No	Solid and Liquid (Citrus based)	Sinus arrest, AV block	PPM	Yes
Lipar et al. 2018/ [9]	49/F	Syncope	Post whiplash neck injury	Solid and Liquid	–	PPM	Yes
Van Damme et al. 2017/ [10]	39/M	Syncope	No	Solid and Liquid	3rd degree AV block	PPM	–
Aydogdu et al. 2017/ [11]	51/F	Presyncope, Syncope	No	Solid food	AV block	Rejected PPM	–
	65/F	Syncope	–	Liquid (Carbonated)	Sinus arrest, 3rd degree AV block	PPM	Yes
	39/F	Presyncope, Syncope	No	Solid food	3rd degree AV block	PPM	Yes
	53/F	Presyncope, Syncope	No	Solid food	Asystole	Diet modification	–
	68/M	Presyncope, Syncope	Atrial Fibrillation	Liquids	Asystole	PPM	Yes
Patel et al. 2017/ [12]	48/M	Syncope, Nausea, Tunnel vision	Hiatus Hernia	Solid and Liquid	Sinus arrest	Hiatus hernia repair surgery	Yes
Zaid et al. 2017/ [13]	71/M	Syncope	Achalasia	Solid food	AV block	–	–
Bhogal et al. 2017/ [14]	68/F	Presyncope	Hiatus Hernia, Metabolic Diseases	Solid food	Sinus Bradycardia, 1st degree AV block	Discontinuation of metoprolol + Proton Pump Inhibitor	No
	59/M	Pre-syncope & Syncope	No	Liquid diet	Premature atrial complexes & Asystole	PPM	Yes
Trinco et al. 2016/ [15]	83/ M	Syncope	Carotid endarterectomy, Metabolic diseases	Solid and Liquid	Sinus bradycardia, 3rd degree AV block	PPM	Yes
Islam et al. 2016/ [16]	60/ F	Presyncope, Syncope	No	Solid food (Large chunk of bread)	AV block	Avoidance of trigger	Yes
Chhetri et al. 2016/ [17]	71/M	Syncope	Fundoplication for GERD	Solid and Liquid (Fizzy drink)	Sinus arrest	PPM	NM
Tiffany et al. 2016/ [18]	80/F	Syncope, palpation, facial flushing	Metabolic diseases, Hypothyrodism	Solid and Liquid	Atrial Tachycardia	Catheter ablation	Yes
Manu et al. 2016/ [19]	13/F	Syncope	Superior sinus atrial septal defect	Solid and Liquid	3rd degree AV block	PPM	Yes
Aaberg et al. 2015/ [20]	62/M	Pre-syncope, Syncope	No	Solid and Liquid	2nd and 3rd degree AV block	PPM	Yes
Kahn et al. 2015/ [4]	89/M	Syncope	Transient Oesophageal dysmotility, Coronary artery diseases	Solid and Liquid (Carbonated)	1st and 2nd degree AV block	PPM	Yes
Saitoh et al. 2015/ [21]	70/M	Syncope	No	Solid food	Asystole	PPM	Yes
Erdogan et al. 2015/ [22]	47/M	Syncope	Achalasia	Solid and Liquid	AV block, Asystole	Pneumatic dilation	Yes
Shashank et al. 2014/ [23]	31/F	Presyncope & Syncope	No	Liquid (Carbonated)	Sinus bradycardia, Asystole	PPM	Yes
	78/ M	Presyncope	Sick sinus syndrome, Metabolic diseases	Solid food	–	PPM + Coffee before meals	Yes
	80/M	Presyncope, Syncope	Hiatus Hernia AF, various cardiac comorbid	Solid food (Sticky food)	–	Avoidance of trigger	Yes
Shah et al. 2014/ [24]	57/M	Presyncope, Syncope	No	Swallow +Cold drink	Advanced heart block for 3–4 s	PPM	Yes
Witcik et al. 2014/ [25]	70/M	Syncope, Weakness, Flushing	Mild AV regurgitation	Liquid (Carbonated)	Atrial Fibrillation with ventricular pause	PPM	Yes
Arihide et al. 2014/ [26]	79/M	Syncope	Coronary artery disease, Metabolic diseases	Solid and Liquid	Sinus arrest	PPM	Yes
Moore et al. 2013/ [27]	65/F	Presyncope, Syncope	No	Solid food	AV block	PPM	Yes
Lambiris et al. 2013/ [28]	54/M	Presyncope, Shortness of breath	No	Solid and Liquid	1st degree AV block	PPM	Yes
Rezvani et al. 2013/ [29]	51/F	Syncope	Post Laparoscopic gastrectomy	Solid and Liquid	AV block	Atropine	Yes
Kim eat al. 2012/ [30]	39/M	Syncope, Chest tightness	No	Liquid (Cold)	3rd degree AV block	Avoidance of trigger	Yes
Knopke et al. 2012/ [31]	49/F	Syncope, Dysphagia, Regurgitation	Hiatus hernia, Diffuse oesophageal spasm	Solid food	3rd degree AV block	PPM	Yes
Foreman et al. 2011/ [32]	52/F	Presyncope, Chest pain	No	Solid food	2nd degree AV block	PPM	Yes
Vanerio et at. 2011/ [33]	84/F	Syncope	Hiatus Hernia	Solid and Liquid (Carbonated)	–	Nissen’s Fundoplication	Yes
Mitra et al. 2011/ [34]	60/F	Presyncope, Syncope	Metabolic Diseases	Solid food	Sinus Bradycardia, 3rd degree AV block	PPM	Yes
Marina et al. 2010/ [35]	37/M	Syncope	Megaoesophagus, Extra Cardiac mass compressing left atrium	Solid and Liquid	–	Deflation of gastric band	–
GY Lee et al. 2010/ [36]	62/M	Syncope, Dysphagia	Atrial Fibrillation, Metabolic diseases	Liquid	Asystole	PPM	Yes
Endean et al. 2010/ [37]	61/ M	Syncope, Chest pain, Vision lost	Post Carotid entaterectomy	Solid food	–	Glycopyrrolate	Yes
Casella et al. 2009/ [38]	66/ M	Syncope	Oesophageal dysmotility, Sick sinus syndrome	Liquid only	AV block	PPM	Yes
Karamitsos et al. 2009/ [39]	82/F	Syncope	Hiatus hernia	Large meal	NM	–	–
Favaretto et al. 2008/ [40]	63/M	Syncope, Odynophagia	Hiatus hernia	Solid and Liquid	Asystole	PPM	Yes
Bajwa et al. 2008/ [41]	51/M	Presyncope, Syncope	Metabolic diseases, Inflammatory bowel diseases	Solid food	Atrial & Ventricular atopic beat	PPM	Yes
Christopher et al. 2008/ [42]	25/F	Syncope	No	Solid and Liquid	3rd degree AV block	PPM	Yes
Fahrner et al. 2008/ [43]	75/M	Syncope	No	Solid and Liquid	AV block	–	–
Patsilinakos et al. 2007/ [44]	86/F	Syncope	Oesophageal stenosis, Ascending aorta aneurysm, Hypothyroidism	Solid and Liquid	Sinus arrest	Avoidance of trigger	Yes
Tuzcu et al. 2007/ [45]	16/F	Syncope, Visual disturbance	No	Solid food	3rd degree AV block, Asystole	PPM	Yes
Omni et al. 2006/ [2]	66/F	Syncope	Metabolic Diseases	Liquid	AV block	PPM	Yes
Gawrieh et al. 2005/ [46]	63/M	Presyncope, Syncope, Dysphagia	Hiatus Hernia	Solid food	AV block, Asystole	PPM	Yes
	63/M	Presyncope, Syncope	Hiatus hernia, Coronary artery diseases, Metabolic diseases	Solid and Liquid	–	Refuse treatment	–
	62/F	Presyncope, Syncope, Dysphagia	Nutcracker oesophagus, Coronary artery diseases	Solid and Liquid	Sinus bradycardia, Sinus arrest	PPM	Yes
Turan et al. 2005/ [47], Kang et al. 2005/ [48]	48/M	Syncope, Dysphagia	Achalasia	Solid food	Sinus bradycardia	PPM	Yes
	59/ M	Syncope	Metabolic diseases	Solid and Liquid	Sinus bradycardia	PPM	–
	59/M	Syncope, Dysphagia	Compression fracture thoracic spine, Graves diseases	Solid food	Sinus bradycardia	Diet habit modification	–
Sreekant et al. 2004/ [49]	85/M	Syncope	Coronary artery diseases, Peripheral vascular diseases	Solid and liquid	Asystole	PPM	Yes
	61/ F	Presyncope	Metabolic diseases	Liquid (Carbonated)	Sinus Bradycardia	–	–
Yoshifumi et al. 2004/ [50]	76/F	Syncope	Hiatus hernia	Solid food	–	–	–
Srivathsan et al. 2003/ [51]	26/M	Presyncope	No	Solid food	Systole	PPM	Yes
Mekawa et al. 2002/ [52]	76/ F	Syncope	Hiatus hernia	Solid and liquid	–	Hernia repair surgery	Yes
Gordon et al. 2002/ [53]	26/F	Syncope, Central chest discomfort	Hiatus hernia	Solid and liquid	Paroxysmal Atrial fibrillation, Ventricle atopic beat	Diet habit modification	Yes
Takeshi et al. 2002 [54]	69/F	Presyncope, Syncope	Metabolic diseases	Solid food	Sinus arrest	–	–
Rasmi et al. 2001/ [55]	16/M	Syncope	No	Liquid (Carbonated)	Asystole	PPM	Yes
Haumer et al. 2000/ [56]	67/ M	Syncope	Coronary artery disease	Liquid	Sinus arrest	Temporary Pacemaker	Yes
Kakuchi et al. 2000/ [57]	21/M	Syncope	Vasovagal syncope	Solid and liquid	AV block	PPM	–
Kazushi et al. 1999/ [58]	69/M	Syncope, Facial flushing, Profuse diarrhoea	Metabolic disease, Stroke	Solid food	–	Cessation of Enalapril	Yes
Olshasky et al. 1999/ [59]	72/M	Presyncope, Syncope	–	Liquid (Cold carbonated)	Sinus bradycardia	PPM	–
Dante et al. 1997/ [60]	78/M	Syncope	Oesophageal carcinoma	Solid food	AV block, Asystole	PPM	Yes
Bellori et al. 1992/ [61]	69/M	Syncope	–	Liquid	Sinus arrest	–	–
SY AO et al. 1991/ [5]	70/M	Incidental	Lung carcinoma	Solid and Liquid	High grade AV block	Atropine before meal	Yes
Shapira et al. 1991/ [62]	63/M	Presyncope, Syncope	Hiatus hernia, Coronary artery disease	Solid and Liquid	2nd degree AV block	PPM	Yes
Kunimoto et al. 1990/ [63]	65/M	Presyncope, Syncope	No	Liquid (Cold)	2nd degree AV block, Asystole	PPM	Yes
Elam et al. 1989/ [64]	44/M	Syncope	No	Solid and Liquid	3rd degree AV block	PPM	Yes
Engelharbt et al. 1986/ [3]	5/F	Syncope	No	Solid and Liquid/ Brush teeth	3rd degree AV block	Close Observation	Yes
Ausubel et al. 1987/ [65]	26/M	Syncope	Heart murmur	Solid food	Sinus bradycardia, AV block	PPM	Yes
Nakano et al. 1987/ [66]	67/M	Syncope, Retrosternal discomfort	Aneurysm descending thoracic aorta	Solid and Liquid	Sinus bradycardia, Sinus arrest	Atropine before meal	Yes
Nakagawa et al. 1987/ [67], Guberman et al.1986/ [68]	48/M	Syncope	No	Solid and Liquid	AV block	Atropine	–
	62/F	Syncope	No	Oesophageal balloon inflation	2nd degree heart block	Propanthelene bromide	No
	62/M	Syncope	Congestive heart failure	Solid food	2nd degree heart block	Discontinuation of digoxin	Yes
Alan et al. 1986/ [69]	56/M	Syncope	Inferior myocardial infarction	Liquid	1st degree heart block	PPM	Yes
Golf et al. 1986/ [70]	15/ F	Syncope	No	Solid and Liquid	SA node blockade with junctional escape rhythm	–	–
Armstrong et al. 1985/ [71]	53/F	Syncope, Dyspnoea, Retrosternal discomfort	Hiatus hernia	Liquid	Sinus bradycardia	PPM	Yes
	58/F	Syncope, Pulseless, Apnoea	Myocardial infarction, Atrial Fibrillation, Stroke	Solid and Liquid	Sinus bradycardia and Asystole	PPM	No
	58/F	Presyncope	No	Solid and Liquid	3rd degree AV block and Asystole	PPM	Yes
	81/F	Syncope	Hiatus hernia, Metabolic disease	Liquid (Hot)	Sinus bradycardia	PPM	Yes
	53/M	Syncope	Myocardial infarction	Liquid (Cold)	2nd degree AV block	PPM	Yes
Kunis et al. 1985/ [72]	60/M	Presyncope, Syncope, Chest pain	Metabolic diseases	Solid food (Hot)	3rd degree AV block, Asystole	PPM	Yes
Drake et al. 1985/ [73]	76/F	Syncope	Myocardial infarction, Metabolic disease	Sight of food	3rd degree AV block	PPM	Yes
Mauro et al. 1985/ [74]	65/ F	Presyncope, syncope	Myocardial ischemia	Solid and Liquid	2nd degree AV block	Atropine	No
Golf et al. 1977 [75]	−/ M	Syncope, Convulsion	No	Solid and Liquid	2nd degree AV block	PPM	Yes
Weaddington et al. 1975/ [76]	71/M	Syncope	Hiatus hernia, Oesophagus carcinoma, Atrial Fibrillation	Solid food	Sinus bradycardia and Asystole	Surgical removal of Oesophageal Carcinoma	Yes
B Wik et al. 1975/ [77]	43/ M	Syncope, Retrosternal chest pain	Rheumatic heart diseases	Liquid (Carbonated)	AV block	PPM	–
Poul et al. 1973/ [78]	64/ F	Syncope	Hiatus hernia, Abnormal oesophageal motility	Solid and Liquid	Sinus bradycardia, AV block	Hernia Repair	Yes
Edgar et al. 1972/ [79]	84/M	Syncope	Hiatus hernia, Metabolic diseases	Solid and Liquid	2nd degree AV block	Atropine	Yes
Keith et al. 1971/ [80]	45/M	Syncope, Dysphagia, Heart burn	Hiatus hernia, Oesophageal stricture	Solid and Liquid	Sinus bradycardia	Dilation of oesophageal stricture	Yes
Rajendra et al. 1971/ [81]	29/ F	Syncope	No	Solid and Liquid	Asystole	Surgical cauterization vagal nerve	Yes
Edgardo et al. 1970/ [82]	73/M	Syncope, Chest pain	Myocardial infarction, Metabolic disease	Solid and Liquid	AV block, Asystole	Atropine	Yes
R P Sapru et al.1968/ [83]	29/F	Presyncope	No	Solid and Liquid	AV block, Asystole	Atropine	Yes
George et al. 1958/ [84]	−/−	Syncope	No	Liquid	–	Discontinuation of digitalis	Yes
Correll et al. 1949/ [85]	67/M	Syncope, Chocking sensation	Oesophageal diverticulum, Digitalis medication	Solid and Liquid	3rd degree AV block	Atropine	Yes

*F* Female, *M* Male, (−) Not Stated, *AV* Atrioventricular, *PPM* Permanent Pacemaker

Pacemaker implantation is the most popular treatment modality. More than half of the patients (55.5%, *n* = 56) were treated with a permanent pacemaker. Almost all (98.1%, *n* = 52) of the patients treated with pacemakers reported resolution of syncopal symptoms. One patient passed away shortly following a PPM implant due to asystole despite a reportedly normal functioning pacemaker [71]. Treatment of an underlying causative factor (15.8%, *n* = 16) was the second most common treatment modality. Treatment of an underlying gastrointestinal disorder has been shown to carry a good likelihood of resolving the swallow syncope. For example, all four cases of hiatal hernia that were corrected surgically had a complete resolution of the swallow syncope. Likewise, dilatation of an oesophageal stricture and an achalasia resulted in complete resolution of swallow syncope. Other reported successful treatments of underlying gastrointestinal diseases included surgical cauterisation of the vagal nerve, long term proton pump inhibitors and surgical excision of an oesophageal carcinoma. Pharmacological management was the preferred treatment option in the 19th and early twentieth century prior to the era of pacemakers. From the limited numbers, atropine was the most widely used, with about 90% efficacy. Table 2.

**Table 2:** Characteristics of 101 reviewed cases of swallow syncope

	Frequency (*n*=)	Percentage (%)
Age Group (*n* = 101)		
Childhood/Adolescent [0–19 years]	6	5.9
Younger adults [20–59 years]	37	36.6
Older adults [60 years and above]	56	55.4
Not stated	2	2.0
Gender (*n* = 101)		
Male	60	59.4
Female	40	39.6
Not Stated	1	1.0
Clinical Presentation (*n* = 101)		
Syncope	100	99.0
Dysphagia	12	11.9
Asymptomatic (incidental diagnosis)	1	1.0
Underlying Diseases (*n* = 100)		
Gastrointestinal Diseases	34	33.7
Hiatal Hernia	19	18.8
Achalasia	3	3.0
Esophageal stricture	3	3.0
Cardiac Diseases	33	32.7
Coronary artery diseases	14	13.9
Atrial Fibrillation	5	5.0
Sick Sinus Syndrome	3	3.0
Comorbidities^a^	28	27.7
Trigger Factor (*n* = 101)		
Any (Solid and Liquid)	55	54.5
Solid only	23	22.8
Liquid only	23	22.8
Type of Arrhythmia (*n* = 101)		
Sinus Dysfunction^b^	34	33.7
Atrioventricular Dysfunction^c^	35	34.7
Combination Sinus and AV Dysfunction	16	15.8
Not Stated	13	12.9
Others^d^	3	3.0
Management (*n* = 101)		
Pacemaker Implantation	56	55.5
Pharmacotherapy	11	10.9
Atropine	9	8.9
Treatment of Underlying causative factor	16	15.8
Surgical correction of hiatal hernia	4	4.0
Dilation of achalasia	1	1.0
Dilation of esophageal stricture	1	1.0
Conservative Management	9	8.9
Avoidance trigger/ diet modification	7	6.9
Close observation/ refused treatment	2	2.0
Not Stated	9	8.9
Documented efficacy of resp. treatment	*Effective (n=)*	*Efficacy rate (%)*
Pacemaker (*n* = 53)	52	98.1
Atropine treatment (*n* = 8)	7	87.5
Surgical correction of Hiatal hernia (*n* = 4)	4	100
Dilation of Achalasia (*n* = 1)	1	100
Dilation of esophageal stricture (*n* = 1)	1	100
Avoidance trigger/ diet modification (*n* = 5)	5	100

^a^Comorbidities defined as hypertension or diabetes mellitus or dyslipidemia or obesity or chronic kidney disease

^b^ Sinus Bradycardia, Sinus Arrest, Asystole; ^c^ First, Second, Third degree Atrioventricular block; ^d^ Atrial Tachycardia, Atrial Fibrillation and others

Various mechanisms regarding the pathogenesis of swallow syncope have been postulated.

The most common postulated mechanism is increased and excessive vagal reflex activation during swallowing causing cardio inhibition [86]. During swallowing, the afferent impulses from the oesophageal plexus travel via the vagus nerve to the nucleus solitarius tract in the medulla oblongata. Subsequently, a corresponding signal that regulates involuntary peristalsis will travel down the parasympathetic efferent fibers through the oesophageal branch of the vagus nerve [87]. The presence of reflex arcs between afferent sensory fibers and efferent parasympathetic fibers of the cardiac branch results in inappropriate vagal activation with bradycardia, disturbance to the conduction system and hypotension secondary to vasodilation [27, 88]. The exact mechanism remains to be elucidated, however, excessive parasympathetic stimulation to the heart seems to be the central mechanism. The fact that atropine, a potent anticholinergic agent, prevents bradyarrhythmia effectively in cases of swallow syncope supports the theory of excessive vagal stimulations [5, 29, 66, 79].

Abnormal oesophageal mechanoreceptors have been postulated to be the primary cause of swallow syncope in individuals with underlying structural and functional disorders of the gastrointestinal system. We demonstrated a reproducible cardio-inhibition with balloon inflation in the mid to lower oesophagus in our patient [48, 89]. The bradyarrhythmia was terminated upon deflation of the balloon suggesting that mechanoreceptors in the mid-lower oesophagus may play a role in the pathogenesis of swallow syncope.

**Fig. 4:**
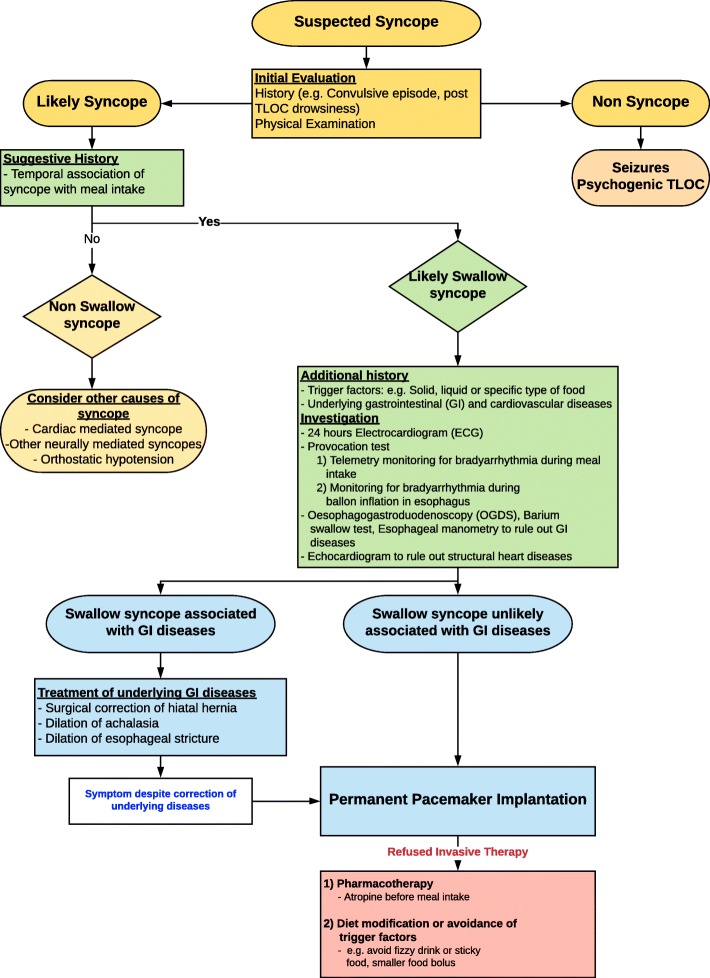
Approach to the diagnostic work-up and management of patients with symptoms suggestive of swallow syncope

Investigations of neurally-mediated syncope should be tailored based on actual precipitants. While a tilt-table test confirmed the presence of a vasovagal response with reproduction of syncope, it did not demonstrate any periods of asystole. The diagnosis in this case was confirmed during OGD with cardiac monitoring and hence investigation with an OGD with haemodynamic monitoring should be considered for individuals with suspected swallow syncope. A diagram depicting a proposed approach to the diagnostic work-up and management of patients with symptoms suggestive of swallow syncope is depicted in (Fig. 4).

## Conclusions

Swallow syncope is a rare cause for syncopal events and should be considered as part of the diagnostic workup. Pacemakers are a safe and efficacious therapeutic option for all patients with that condition. In patients with associated gastrointestinal disease, specific treatment of the underlying disease has a high likelihood of resolving the swallow syncope without the need for permanent pacing.

## Data Availability

The datasets used and/or analysed in the literature review are available from the corresponding author on reasonable request.

## Abbreviations

AV block: Atrioventricular block
ECG: Electrocardiogram
OGD: Oesophagogastroduodenoscopy
PPM: Permanent pacemaker
